# It takes a village: the Multi-Country Multi-City (MCC) Collaborative Research Network

**DOI:** 10.1097/EE9.0000000000000340

**Published:** 2024-09-10

**Authors:** Bert Brunekreef

**Affiliations:** a Institute for Risk Assessment Sciences, Utrecht University, Utrecht, The Netherlands

A forthcoming “special collection” in *Environmental Epidemiology* highlights a series of new findings from the MCC study, the Multi-Country Multi-City (MCC) Collaborative Research Network. For almost 10 years now, this unique collaboration has raised the science of the short-term effects of weather and air pollution on population health to new levels.

Research on the short-term effects of weather and air pollution on population health has a long history. An early report on the effects of very high air pollution levels on mortality describes a smog along the Meuse Valley in Belgium, which occurred in 1930.^1,2^ Interestingly, the original 1936 report almost quantitatively predicted the number of deaths that would occur in London under similar circumstances, which occurred in the famous December 1952 episode that led to the introduction of the first modern air pollution legislation in the world.

Effects of especially high temperatures on health have also been studied for a long time. A remarkable report from 1938^3^ documented a detailed breakdown of heat-related deaths by cause, age, sex, city size, and other modifiers in Massachusetts.

The effects of excessive heat and air pollution on mortality are well documented (and fairly easy to document) but such early studies do not answer the question of whether nonexcessive temperatures and air pollution levels are still associated with mortality and other adverse health outcomes. Efforts to identify a threshold for the association between air pollution and mortality date back at least 40 years.^4^ Such studies require large populations as the effects at low concentrations, if any, are bound to be subtle. They also require sufficient days with low air pollution concentrations, which were rare in days of old when major cities with large enough populations across the world almost all suffered from high pollution levels. Such limitations also apply to studies of population health effects of subtle increases, or decreases, in day-to-day temperatures. And yes, the effects of heat waves and excessively high temperatures have generated more interest in recent decades than the effects of cold temperatures, but these are of equal interest. An early study from the Netherlands^5^ clearly showed that even in a country without very high or low temperature extremes, mortality increased when temperatures were below or above a narrow optimum temperature range of a few degrees Celsius above and below 16.5ºC.

The 1990s saw an explosion of studies on short-term associations between air pollution and mortality. Soon, multicenter studies were organized to overcome the limitations of studying such associations in single cities, including the Air Pollution and Health: a European Approach (APHEA) in Europe,^6^ the National Morbidity, Mortality, and Air Pollution Study (NMMAPS) in the United States,^7^ and a combined European–USA–Canadian effort.^8^

Interest in the acute effects of especially high temperatures strongly increased in the first decade of the 21st century, in response to the 2003 heatwave that killed tens of thousands of Europeans.^9–11^ Climate change is making serious heat waves ever more likely, and interest seems to have focused more on the effects of heat than on the effects of cold. Yet, excess winter mortality due to cold weather may be equally or even more important, especially in countries with mild climates not adapted to severe cold spells.^12^

As warm and cold weather on the one hand and high air pollution concentrations on the other hand tend to covary, an important question is whether their effects on population health can be separated. Early work from the Netherlands suggested air pollution (measured as SO_2_) effects were confounded by temperature effects.^13^ Later work has generally found that the effects of air pollution and weather were both important.^14^

The MCC study, highlighted in the special collection of articles in this volume of *Environmental Epidemiology*, is a (relatively) new kid on this very populous block of studies as they have emerged over the last several decades. It is in many ways a unique enterprise. It is based on the voluntary contributions of many scientists all over the world, who contribute their data and time to make the analyses of huge datasets possible. The MCC study has a much wider geographic coverage than previous multicenter studies, which were largely restricted to Europe and North America, in addition to a number of more modest contributions from South-East Asia.^15^ Over the years, the MCC study has broken new ground both in terms of methodology and topics that were addressed. It has produced many landmark articles, of which the *Lancet* paper on effects of high and low temperatures^16^ and the *New England Journal of Medicine* paper on short-term effects of particulate matter air pollution^17^ are just a few. The articles published in the special collection address topics such as the influence of weather and air pollution on COVID and on economic loss, the modifying role of land use, and the decadal change in minimum mortality temperature related to global warming. A full introduction to the MCC achievements is to be found in the companion paper by Gasparrini and colleagues.^18^ Perhaps the most unique feature of the MCC enterprise is that it is almost completely unfunded. It survives, no, flourishes, because of the trust and companionship among the many authors and collaborating centers, and because of the gentle and continuous leadership of the coordinating investigators. As they say: it takes a village to raise a child (in this case a worldwide village) and the MCC child, now about 10 years old, is doing very well!

## Conflicts of interest statement

The author declares that there is no conflicts of interest with regard to the content of this report.
